# Lifestyle, Sleep, and Psychological Correlates of Social Participation Across Mediterranean and Neighbouring Countries: Findings from the MEDIET4ALL Survey

**DOI:** 10.3390/healthcare14183039

**Published:** 2026-09-16

**Authors:** Achraf Ammar, Khaled Trabelsi, Atef Salem, Maria Antonieta Touriz Bonifaz, Bassem Bouaziz, Mohamed Ali Boujelbane, Mohamed Kerkeni, Liwa Masmoudi, Hadeel Ali Ghazzawi, Adam Tawfiq Amawi, Bekir Erhan Orhan, Raynier Zambrano-Villacres, Juliane Heydenreich, Christiana Schallhorn, Tarak Driss, Giuseppe Grosso, Piotr Zmijewski, Haitham Jahrami, Waqar Husain, Hamdi Chtourou, Wolfgang I. Schöllhorn

**Affiliations:** 1 Department of Training and Movement Science, Institute of Sport Science, Johannes Gutenberg-University Mainz, 55122 Mainz, Germany; asalem@uni-mainz.de (A.S.); wolfgang.schoellhorn@uni-mainz.de (W.I.S.); 2 Research Laboratory, Molecular Bases of Human Pathology, LR19ES13, Faculty of Medicine of Sfax, University of Sfax, Sfax 3000, Tunisia; 3 Interdisciplinary Laboratory in Neurosciences, Physiology, and Psychology: Physical Activity, Health, and Learning (LINP2), UFR STAPS, Paris Nanterre University, 92000 Nanterre, France; tdriss@parisnanterre.fr; 4 Department of Nutrition and Food Technology, School of Agriculture, The University of Jordan, Amman 11942, Jordan; h.ghazzawi@ju.edu.jo; 5 High Institute of Sport and Physical Education of Sfax, University of Sfax, Sfax 3000, Tunisia; trabelsikhaled@gmail.com (K.T.); mboujelb@uni-mainz.de (M.A.B.); mohamed.kerkeni@isseps.usf.tn (M.K.); hamdi.chtourou@isseps.usf.tn (H.C.); 6 Research Laboratory: Education, Motricity, Sport and Health, EM2S, LR19JS01, High Institute of Sport and Physical Education of Sfax, University of Sfax, Sfax 3000, Tunisia; liwa.masmoudi@isseps.usf.tn; 7 Department of Movement Sciences and Sports Training, School of Sports Sciences, The University of Jordan, Amman 11942, Jordan; a.amawi@ju.edu.jo; 8 Facultad de Ciencias de la Salud, Universidad Católica de Santiago de Guayaquil, Av. Pdte. Carlos Julio Arosemena Tola, Guayaquil 090615, Ecuador; maria.touriz@cu.ucsg.edu.ec; 9 Facultad de Ciencias Médicas, Universidad de Guayaquil, Avenida 10 NO, Guayaquil 090613, Ecuador; 10 Multimedia InfoRmation Systems and Advanced Computing Laboratory (MIRACL), University of Sfax, Sfax 3000, Tunisia; bassem.bouaziz@isims.usf.tn; 11 Higher Institute of Computer Science and Multimedia of Sfax (ISIMS), University of Sfax, Sfax 3000, Tunisia; 12 Faculty of Sports Sciences, Istanbul Aydın University, Istanbul 34295, Türkiye; bekirerhanorhan@aydin.edu.tr; 13 Facultad de Ciencias de la Salud y Desarrollo Humano, Universidad Ecotec, Km. 13.5 Samborondón, Samborondón EC092302, Ecuador; razambrano@ecotec.edu.ec; 14 Department of Experimental Sports Nutrition, Faculty of Sports Sciences, Leipzig University, 04109 Leipzig, Germany; juliane.heydenreich@uni-leipzig.de; 15 Department of Sports Economics, Sociology and History, Institute of Sport Science, Johannes Gutenberg-University Mainz, 55122 Mainz, Germany; christiana.schallhorn@uni-mainz.de; 16 Department of Biomedical and Biotechnological Sciences, University of Catania, 95123 Catania, Italy; giuseppe.grosso@unict.it; 17 Department of Biochemistry, Gdansk University of Physical Education and Sport, 80-336 Gdansk, Poland; piotr.zmijewski@insp.waw.pl; 18 Government Hospitals, Manama 329, Bahrain; hjahrami@hospitals.gov.bh; 19 Department of Psychiatry, College of Medicine and Health Sciences, Arabian Gulf University, Manama 329, Bahrain; 20 Department of Humanities, COMSATS University Islamabad, Islamabad 45550, Pakistan; drsukoon@gmail.com; 21 Research Unit, Physical Activity, Sport, and Health, UR18JS01, National Observatory of Sport, Tunis 1003, Tunisia

**Keywords:** social participation, lifestyle, Mediterranean lifestyle, psychological well-being, life satisfaction, physical activity, sedentary behaviour, sleep health, social health, MEDIET4ALL

## Abstract

**Background:** Social participation is an important psychosocial dimension of health, but its associations with multiple lifestyle and psychological domains have rarely been examined simultaneously across Mediterranean and neighbouring countries. This study examined sociodemographic, health-related, Mediterranean dietary, movement-related, sleep-related, psychological, and technology-use correlates of social participation. **Methods:** Data were analysed from 4010 adults participating in the multinational MEDIET4ALL survey across ten countries. Social participation was assessed using the Short Social Participation Questionnaire (SSPQ). Hierarchical multiple linear regression examined associations across these domains, with the fully adjusted Model 6 designated as the primary inferential model. Sensitivity analyses examined sex- and region-related effect modification, country fixed effects, and categorical-variable coding. Exploratory indirect associations involving selected lifestyle behaviours and life satisfaction were estimated using bootstrapping. **Results:** The fully adjusted Model 6 explained 23% of the variance in social participation (adjusted R^2^ = 0.224). The largest standardized associations among the statistically supported correlates were observed for age (β = −0.224), anxiety (β = 0.194), life satisfaction (β = 0.160), physical activity (β = 0.152), and depression (β = −0.149). Dietary associations were generally comparable across sexes, but the positive association involving Mediterranean dietary habits was stronger among participants from Mediterranean countries (interaction *p* = 0.002). The principal associations were materially consistent in country fixed-effects and categorical-coding sensitivity analyses. Exploratory bootstrapped analyses identified statistically supported indirect associations through life satisfaction for both Mediterranean dietary dimensions and physical activity in relation to higher social participation and for sitting time in relation to lower social participation. **Conclusions:** Social participation was associated with multiple lifestyle, psychological, sleep-related, and sociodemographic domains within the MEDIET4ALL sample. These findings are associative, while the regional and indirect-association findings are exploratory and hypothesis-generating. Given the cross-sectional design, the results establish neither temporal nor causal relationships. Longitudinal and intervention studies are required to determine directionality and practical relevance.

## 1. Introduction

Social participation is increasingly recognized as a central determinant of health, well-being, and healthy ageing, reflecting individuals’ engagement in interpersonal, community, cultural, and everyday social activities [1,2,3]. Beyond representing a social behaviour in itself, social participation is closely linked to broader constructs such as social integration, social connection, perceived belonging, and access to supportive environments [4,5,6]. These dimensions may influence health through multiple pathways, including emotional support, behavioural activation, stress regulation, reinforcement of healthy routines, and opportunities for shared lifestyle practices [4,5,7]. Conversely, lower social participation and related forms of social disconnection, including social isolation and loneliness, have been associated with poorer mental health, lower well-being, functional decline, and increased morbidity and mortality risk [1,3,8,9,10,11]. Accordingly, understanding the behavioural, psychosocial, and lifestyle correlates of social participation is important for developing integrated public-health strategies that address not only individual behaviours but also the social environments in which such behaviours are adopted and maintained.

The Mediterranean lifestyle provides a particularly relevant framework for examining social participation because it conceptualizes health-promoting behaviour as a multidimensional pattern rather than as diet alone, integrating dietary choices with meal practices, physical activity, rest, and social interaction [12,13,14]. The Mediterranean Lifestyle Index (MEDLIFE) captures Mediterranean dietary consumption patterns, Mediterranean dietary habits, and broader lifestyle behaviours including physical activity, rest, social interaction, and conviviality [12,13]. This broader perspective is important because traditional Mediterranean living is embedded in shared meals, family and community interactions, active routines, and culturally shaped daily practices [15,16,17]. However, research has focused predominantly on dietary adherence and health outcomes, with comparatively less attention to social participation as a distinct outcome within the Mediterranean-lifestyle framework [12,15,17]. This is an important limitation, because social participation may both reflect and facilitate Mediterranean lifestyle adherence through collective food practices, active leisure, community engagement, psychosocial support, and shared lifestyle routines [12,17,18,19].

Social participation is also interrelated with several behavioural and psychosocial domains, including physical activity, sedentary behaviour, sleep health, and psychological well-being [4,10,20,21]. Physical activity and sedentary behaviour are particularly relevant because they are shaped not only by individual motivation but also by social opportunities, interpersonal support, built environments, and daily routines [20,22,23]. Regular physical activity may promote social participation by increasing opportunities for social contact through sport, active commuting, group-based leisure, and shared recreational contexts, whereas social engagement may facilitate sustained physical activity through motivation, accountability, support, and shared behavioural norms [20,21,23]. Conversely, prolonged sedentary behaviour may reduce opportunities for interpersonal interaction and may cluster with poorer psychosocial well-being, lower behavioural activation, and adverse health outcomes [24,25,26,27]. Sleep health may also play a role, as poor sleep quality, prolonged sleep latency, low sleep efficiency, and insomnia symptoms are associated with fatigue, emotional dysregulation, impaired daytime functioning, and poorer mental health, all of which may constrain social engagement [28,29,30,31]. At the same time, social participation, social support, and perceived connectedness may support sleep through stress-buffering mechanisms, emotional support, and more structured daily routines [4,32,33]. These potentially reinforcing relationships highlight the need to examine social participation alongside movement behaviours, sedentary time, and sleep indicators rather than as an isolated psychosocial variable [4,21,34].

Psychological well-being and distress are also closely linked with social participation. Life satisfaction reflects a global cognitive evaluation of one’s life circumstances and is widely used as a positive indicator of subjective well-being and mental health [35,36,37]. Higher life satisfaction may facilitate social participation by increasing motivation, perceived self-efficacy, openness to social interaction, and engagement in meaningful activities, whereas social participation may enhance life satisfaction through belonging, support, role fulfilment, and social integration [3,4,6,33]. Psychological distress, including depression, anxiety, and stress symptoms, may also shape social participation, although associations may differ by symptom domain and sociocultural context [10,38,39]. Depressive symptoms may reduce social engagement through anhedonia, low energy, social withdrawal, and reduced motivation, whereas anxiety and stress may show more complex associations depending on interpersonal demands, coping strategies, social obligations, and cultural norms [10,39,40]. These relationships are especially relevant in multinational samples, where social participation may be influenced not only by individual psychological status but also by broader sociocultural norms, family structures, urbanization, community infrastructure, and country-specific lifestyle traditions [4,22,41,42,43].

The MEDIET4ALL project provides an opportunity to address this gap in a multinational sample from Mediterranean and neighbouring countries. Previous MEDIET4ALL analyses identified regional, sex-related, and cross-country variation in Mediterranean lifestyle adherence and social participation and demonstrated interrelationships among dietary, movement-related, sleep-related, psychological, and health domains [19,34,43,44,45,46,47,48]. However, these studies did not examine social participation as the primary outcome within a single multidomain model. More broadly, social participation has generally been considered as an exposure, contextual factor, or component of lifestyle profiles, while comparatively little multinational evidence has examined Mediterranean dietary behaviours, physical activity, sedentary time, sleep characteristics, positive well-being, and psychological distress simultaneously in relation to social participation.

Social participation was selected as the primary outcome because social interaction and conviviality are recognized components of the Mediterranean lifestyle, yet comparatively little research has examined social participation as a distinct psychosocial outcome within this framework. Importantly, the present analysis used only the Mediterranean dietary consumption-pattern and dietary-habits domains of MEDLIFE as predictors. The third MEDLIFE domain, which contains social-interaction and conviviality items, was excluded to reduce direct construct overlap with the SSPQ and enable social participation to be examined as a distinct outcome. Identifying its multidomain correlates may help inform socially embedded and context-sensitive approaches to Mediterranean lifestyle promotion. On the other hand, Life satisfaction was selected as the linking construct for the exploratory indirect-association analyses because it captures positive evaluative well-being, is conceptually distinct from psychological distress, and has been associated with social connectedness and multiple lifestyle behaviours [4,21,35,49]. Restricting these analyses to life satisfaction maintained conceptual focus and limited extensive data-driven testing of alternative psychological constructs.

Accordingly, the primary objective was to examine the sociodemographic, health-related, Mediterranean dietary, movement-related, sleep-related, psychological, and technology-use correlates of social participation in the multinational MEDIET4ALL sample. We hypothesized that stronger Mediterranean dietary adherence, greater physical activity, lower sitting time, more favourable sleep characteristics, higher life satisfaction, and lower psychological distress would be associated with greater social participation. As a secondary exploratory objective, we examined a restricted set of statistical indirect associations involving the two Mediterranean dietary dimensions, physical activity, sitting time, life satisfaction, and social participation. These secondary analyses were hypothesis-generating and were not intended to establish temporal ordering or causal mediation.

## 2. Materials and Methods

### 2.1. Study Design and Participants

The present cross-sectional analysis used data from MEDIET4ALL, an international survey of Mediterranean lifestyle practices and associated behavioural, psychological, social, health-related, and sociodemographic characteristics. Data collection extended for approximately four months from summer 2024 and covered Germany, France, Italy, Spain, Luxembourg, Tunisia, Algeria, Morocco, Türkiye, and Jordan.

Adults were invited through an open, non-probability recruitment strategy. Survey invitations were disseminated through the participating institutions and consortium networks, email and professional mailing networks, institutional webpages, and social media channels. Invitations could also be redistributed within participating national networks. Neither probability-based population sampling nor country-specific recruitment quotas were used. The sample should consequently be understood as a multinational analytical sample and not as representative of the adult populations of the participating countries. Eligibility required an age of at least 18 years, residence in one of the ten participating countries, ability to complete an available language version, and provision of electronic informed consent. Individuals reporting a diagnosis of cognitive impairment were not eligible. Participation was voluntary and anonymous.

The questionnaire was delivered through the GDPR-compliant SoSci Survey infrastructure hosted by Johannes Gutenberg University Mainz, Germany. Respondents could select English, German, French, Italian, Spanish, Arabic, or Turkish. When an established version of an instrument was unavailable in a required language, forward–backward translation and multilingual expert review were undertaken in accordance with cross-cultural adaptation guidance [50]. The complete multilingual survey was also pilot-tested before dissemination. Test–retest correlations for translated survey components ranged from 0.81 to 0.94 in the MEDIET4ALL methodological evaluation [19,34,43,44,45,51]. These procedures supported multilingual administration but did not constitute formal measurement-invariance testing.

More than 8000 survey entries were initially recorded. Retention required a finalized questionnaire and satisfaction of predefined eligibility and data-quality criteria. Partial submissions and entries without valid consent were excluded. Screening also addressed duplicate or near-duplicate records using IP-address concordance, timestamp proximity, demographic similarity, and response patterns; logical inconsistencies across related questions; and implausible anthropometric, movement-related, or sleep-related values. Because an entry could fail more than one criterion, exclusion categories were not treated as mutually exclusive. Following this process, 4010 valid and complete responses were retained. The retained dataset contained no missing values for the SSPQ outcome or the variables included in the fully adjusted Model 6. All 4010 participants were therefore included in that analysis, and no statistical imputation was performed. Further details of survey development and data-quality procedures have been reported previously [19,34,43,44,45,51].

The study followed the Declaration of Helsinki and was approved by the Ethics Committee of the Faculty of Medicine, University of Sfax (Approval Code: 058/24).

### 2.2. Survey Instruments and Measured Variables

The questionnaire combined established or previously applied self-report measures covering social participation, Mediterranean dietary practices, movement behaviours, sleep, psychological functioning, technology use, and relevant background characteristics.

#### 2.2.1. Sociodemographic and Health-Related Characteristics

Participants reported age, sex, country of residence, educational attainment, employment status, marital status, and whether they lived in an urban, suburban, or rural environment. Smoking, alcohol consumption, and perceived health status were also recorded. Body mass index (BMI; kg/m^2^) was calculated from self-reported weight and height. These variables were included as background covariates because of their potential relationships with both lifestyle behaviours and social participation.

For regional analyses, France, Italy, Spain, Tunisia, Algeria, Morocco, and Türkiye were classified as Mediterranean countries, while Germany, Luxembourg, and Jordan formed the neighbouring/non-Mediterranean comparison group, consistent with the prespecified MEDIET4ALL classification. This grouping represented a broad contextual comparison and was not intended to imply cultural or socioeconomic homogeneity within either group.

#### 2.2.2. Social Participation

The primary outcome was the total score from the 14-item Short Social Participation Questionnaire (SSPQ), an adaptation informed by the parent Social Participation Questionnaire. The 22-item parent instrument was evaluated using Rasch analysis in 789 adults with depressive symptoms and demonstrated support for internal validity and unidimensional measurement after response-category rescoring, with a Person Separation Index of approximately 0.72, indicating reasonable reliability for group comparisons [52]. The 14-item format had previously been applied in the international ECLB-COVID19 survey following pilot testing and multilingual adaptation procedures [53]. It was selected for MEDIET4ALL to reduce respondent burden within a survey covering numerous health and lifestyle domains.

The first ten SSPQ items assess the frequency of interpersonal and social activities on a five-point scale from 1 (“never”) to 5 (“all the time”). Four additional community-participation items use “no” and “yes” responses, scored 1 and 5, respectively. Scores are summed across the 14 items and range from 14 to 70; higher totals represent greater reported social participation.

Using the present dataset, the scale showed acceptable overall internal consistency (Cronbach’s α = 0.76; McDonald’s ω = 0.78). Across the seven questionnaire languages, α ranged from 0.60 to 0.80 and ω from 0.63 to 0.83, indicating some variation among language groups. These results provide preliminary reliability information only. They do not demonstrate cross-language equivalence, and formal multilingual measurement-invariance testing was not undertaken.

#### 2.2.3. Mediterranean Lifestyle Adherence

Mediterranean dietary behaviours were derived from the Mediterranean Lifestyle Index (MEDLIFE), which comprises three domains: Mediterranean dietary consumption patterns, Mediterranean dietary habits, and a broader lifestyle domain covering physical activity, rest, social interaction, and conviviality [12]. Items were scored using the published procedure, with larger domain scores indicating closer adherence to the corresponding Mediterranean practices.

Only the dietary consumption-pattern and dietary-habits domains were entered as predictors. The third domain was not used because it contains social-interaction and conviviality items that overlap conceptually with the social-participation outcome. Separating the two dietary domains also allowed food-consumption patterns and behaviourally oriented eating practices to be examined without incorporating social content into the predictor.

#### 2.2.4. Physical Activity and Sedentary Behaviour

Weekly physical activity was estimated with the short form of the International Physical Activity Questionnaire (IPAQ-SF) [54]. Reported walking and moderate- and vigorous-intensity activity were converted into metabolic-equivalent minutes per week (MET-min/week). Because these values were positively skewed, regression analyses used ln(MET-min/week + 1) consistent with the approach used in the MEDIET4ALL physical activity and sedentary behaviour paper [46]. Participants also reported their usual daily sitting time, which was analysed continuously as a separate indicator of sedentary behaviour.

#### 2.2.5. Sleep Characteristics and Insomnia Severity

Sleep was represented by habitual sleep duration, sleep latency, sleep efficiency, subjective sleep quality, and insomnia severity. The first four indicators were obtained from questions adapted from established sleep-assessment frameworks, including the Pittsburgh Sleep Quality Index. Sleep efficiency was calculated as follows:

Sleep efficiency (%) = (reported sleep duration/time spent in bed) × 100.

Subjective sleep quality was recorded on four ordered levels from 1 (“very bad”) to 4 (“very good”), so higher values indicated better perceived sleep. Insomnia symptoms were assessed using the seven-item Insomnia Severity Index (ISI) [55]. ISI items are scored from 0 to 4 and summed to produce a total from 0 to 28, with higher scores reflecting greater insomnia severity.

#### 2.2.6. Psychological Well-Being and Distress

Life satisfaction was measured with the three-item Short Life Satisfaction Questionnaire (SLSQ), which was developed from the conceptual framework of the Satisfaction with Life Scale [35]. Each statement is rated from 1 (“strongly disagree”) to 7 (“strongly agree”). The summed score ranges from 3 to 21, with higher values indicating greater life satisfaction.

Depressive, anxiety, and stress symptoms during the preceding week were assessed using the 21-item Depression Anxiety Stress Scales (DASS-21) [38]. Each of its three subscales contains seven items rated from 0 (“did not apply to me”) to 3 (“applied to me very much or most of the time”). Higher subscale scores denote greater symptom burden.

#### 2.2.7. Technology Use

The Short Technology-Use Questionnaire (STuQL) contains three items addressing how frequently technology is used in connection with social participation, dietary practices, and physical activity. Responses range from 1 (“never”) to 5 (“all the time”), producing a total score from 3 to 15. Higher scores indicate more frequent technology use within these lifestyle contexts. The STuQL has been previously applied in confinement-related lifestyle research and earlier MEDIET4ALL analyses.

### 2.3. Statistical Analysis

Descriptive analyses and the primary hierarchical models were conducted using IBM SPSS Statistics, version 29 (IBM Corp., Chicago, IL, USA). Continuous variables were summarized using means and standard deviations, while categorical variables were described using frequencies and percentages.

Hierarchical multiple linear regression was used to examine adjusted correlates of the SSPQ total score. Predictors were organized into conceptually defined blocks so that changes in explained variance could be evaluated as additional domains were introduced. Model 1 contained age, sex, region, education, employment status, marital status, and living environment. Model 2 added BMI, smoking, alcohol consumption, and self-reported health. Model 3 added the MEDLIFE dietary consumption-pattern and dietary-habits domains. Model 4 incorporated ln (MET-min/week + 1) and daily sitting time. Model 5 additionally contained sleep duration, sleep latency, sleep efficiency, subjective sleep quality, and ISI total score. Finally, Model 6 added life satisfaction, depression, anxiety, stress, and STuQL total score. The ordering of these blocks was intended to describe progressive adjustment and domain-specific increments in explained variance, not temporal or causal ordering.

Model 6, which retained the complete prespecified predictor set, was designated as the primary inferential model. An exploratory parsimonious Model 7 was derived by retaining predictors with nominal *p* < 0.05 in Model 6 to provide a concise descriptive summary. Because this reduction was data-driven, Model 7 was not used as the principal basis for inference, and its coefficients and confidence intervals were interpreted as exploratory. Model 6 also served as the reference specification for diagnostic, multiplicity, interaction, and sensitivity analyses.

In the prespecified hierarchical models, binary variables were represented by binary indicators, whereas multi-category sociodemographic and health-related covariates were entered using their survey codes, consistent with previous MEDIET4ALL analyses. To assess whether this coding imposed inappropriate single linear contrasts, Model 6 was re-estimated with education, employment status, marital status, living environment, smoking, alcohol consumption, and self-reported health represented by indicator variables. Reference groups were no schooling completed, employed, single, urban residence, non-smoking, no alcohol consumption, and healthy self-reported status, respectively.

Sensitivity analyses also examined whether associations involving the two MEDLIFE dietary dimensions differed by sex or region. Model 6 was estimated separately within men and women and within Mediterranean and neighbouring/non-Mediterranean groups, omitting the corresponding stratification variable. Formal effect modification was evaluated in pooled models by adding interactions between each mean-centred dietary-domain score and sex or region. Interpretation was based on the interaction coefficient rather than on differences in subgroup-specific statistical significance.

A separate country fixed-effects analysis replaced the broad regional indicator with country-specific indicators while retaining all other Model 6 predictors. This specification accounted for differences in country-specific intercepts and assessed whether the principal individual-level associations were robust to measured and unmeasured characteristics that were constant within countries. It was not intended to estimate country-level causal effects or between-country variance. Given the presence of only ten country units, conventional country-clustered standard errors were not used [56].

The Benjamini–Hochberg false discovery rate procedure was applied across the 25 predictor tests in Model 6. Nominal *p*-values and adjusted q-values are reported, with q < 0.05 interpreted as statistical support after multiplicity adjustment. This procedure was used as a robustness assessment and did not modify the prespecified hierarchical sequence.

Exploratory indirect-association analyses evaluated whether life satisfaction statistically linked either MEDLIFE dietary domain, physical activity, or sitting time with social participation. Life satisfaction was selected because it represents positive evaluative well-being, is conceptually distinct from the psychological-distress variables, and has established relationships with both lifestyle behaviours and social engagement. Restricting the analyses to this construct limited extensive post hoc testing of alternative psychological linking variables. Social participation was specified as the outcome in each model, and adjustment included age, sex, region, education, employment status, BMI, smoking, alcohol consumption, and self-reported health. Physical activity was entered as ln(MET-min/week + 1).

Indirect associations were quantified using 5000 nonparametric bootstrap resamples and 95% confidence intervals. Bootstrapping was used because an indirect association is calculated from the product of component regression coefficients, whose sampling distribution may be asymmetric. Statistical support was inferred when the bootstrap interval excluded zero [57,58]. Because all variables were measured concurrently, these models were interpreted only as exploratory descriptions of indirect statistical relationships. They do not establish mediation, temporal sequence, or causal mechanisms; life satisfaction was therefore regarded as a statistical linking construct rather than a demonstrated causal mediator.

Before interpreting the regression models, linearity and homoscedasticity were assessed using residual-versus-fitted plots, and residual distributions were examined using histograms and normal Q–Q plots. Standardized residuals, leverage values, and Cook’s distance were inspected for potentially influential observations. No individually dominant observation was detected in Model 6 (maximum Cook’s distance = 0.014). Multicollinearity was evaluated using tolerance and variance inflation factors (VIF). Sleep latency and sleep efficiency showed substantial overlap (VIF = 15.44 and 16.69, respectively), whereas the remaining predictors had acceptable values. Model 6 was therefore re-estimated without these two sleep variables; their exclusion produced negligible changes in model fit and in the principal lifestyle and psychological coefficients. All tests were two-sided, with nominal statistical significance defined as *p* < 0.05.

## 3. Results

### 3.1. Descriptive Characteristics of the Study Population

The analytical sample comprised 4010 adults from ten Mediterranean and neighbouring countries, of whom 59.5% were women, 58.8% reported higher educational attainment, 50.7% were employed, and 66.3% lived in urban environments. Mediterranean lifestyle adherence and movement behaviours varied across the sample: 31.1%, 46.8%, and 22.1% of participants were classified as having low, medium, and high Mediterranean lifestyle adherence, respectively, while 60.3%, 20.2%, and 19.5% had low, moderate, and high physical activity levels. Mean daily sitting time was 5.59 ± 3.04 h. Additional sample characteristics have been reported in previous MEDIET4ALL publications [19,34,43,44,45].

### 3.2. Hierarchical Regression Analyses Examining Social Participation

Table 1 presents the hierarchical regression results, with complete estimates for the primary fully adjusted Model 6 and standardized coefficients for the exploratory model 7. Model 6 explained 22.9% of the variance in social participation (R^2^ = 0.229; adjusted R^2^ = 0.224). Across the hierarchical sequence, the largest increase in explained variance occurred when psychological and technology-use variables were added (ΔR^2^ = 0.043), followed by movement behaviours (ΔR^2^ = 0.025), health-related variables (ΔR^2^ = 0.023), Mediterranean dietary variables (ΔR^2^ = 0.018), and sleep-related variables (ΔR^2^ = 0.015). In the primary Model 6, the largest standardized associations were observed for age (β = −0.224), anxiety (β = 0.19), life satisfaction (β = 0.16), physical activity (β = 0.15), and depression (β = −0.15). Among the focal lifestyle and sleep-related variables, both MEDLIFE dietary dimensions daily sitting time, sleep duration, subjective sleep quality, and insomnia severity also remained associated with social participation after full adjustment. Complete Model 6 estimates, including B, 95% CIs, β, and *p*-values, are presented in Table 1. Model 7 retained 16 predictors with nominal *p* < 0.05 in Model 6 and explained a similar proportion of variance (R^2^ = 0.227; adjusted R^2^ = 0.224). Its standardized coefficients are included in the final column of Table 1 solely as an exploratory parsimonious summary and were not used as the primary basis for inference.

### 3.3. Collinearity, Sensitivity, and Robustness Analyses

Collinearity diagnostics for Model 6 are presented in Supplementary Table S1. Most predictors had VIF values below 5, whereas sleep latency (VIF = 15.44) and sleep efficiency (VIF = 16.69) showed substantial collinearity. Re-estimating Model 6 without these variables produced negligible changes in model fit (R^2^ = 0.229 and adjusted R^2^ = 0.224 in both specifications) or in the principal coefficient patterns.

Formal interaction analyses provided no evidence that the association between MEDLIFE dietary consumption patterns and social participation differed by region (interaction B = −0.108, 95% CI: −0.408 to 0.191, *p* = 0.478) or sex (interaction B = 0.066, 95% CI: −0.221 to 0.353, *p* = 0.652). The association involving MEDLIFE dietary habits also did not differ by sex (interaction B = −0.019, 95% CI: −0.380 to 0.342, *p* = 0.917). In contrast, the dietary-habits association differed by region (interaction B = −0.582, 95% CI: −0.949 to −0.216, *p* = 0.002), with a stronger positive association among participants from Mediterranean countries. Subgroup-specific estimates and formal interaction results are reported in Supplementary Table S2. The country fixed-effects specification produced materially consistent estimates for the principal dietary, movement-related, and psychological correlates after replacing the broad regional indicator with country-specific indicators (Supplementary Table S2, Panel D). Re-estimating Model 6 using indicator coding for the multi-category sociodemographic and health-related variables likewise did not materially alter the principal associations (Supplementary Table S3).

Following Benjamini–Hochberg correction across all 25 predictor tests in Model 6, each of the 16 associations with nominal *p* < 0.05 remained statistically supported at q < 0.05. Nominal *p*-values and FDR-adjusted q-values are reported in Supplementary Table S4.

### 3.4. Exploratory Statistical Indirect Associations Involving Life Satisfaction

**Figure 1:**
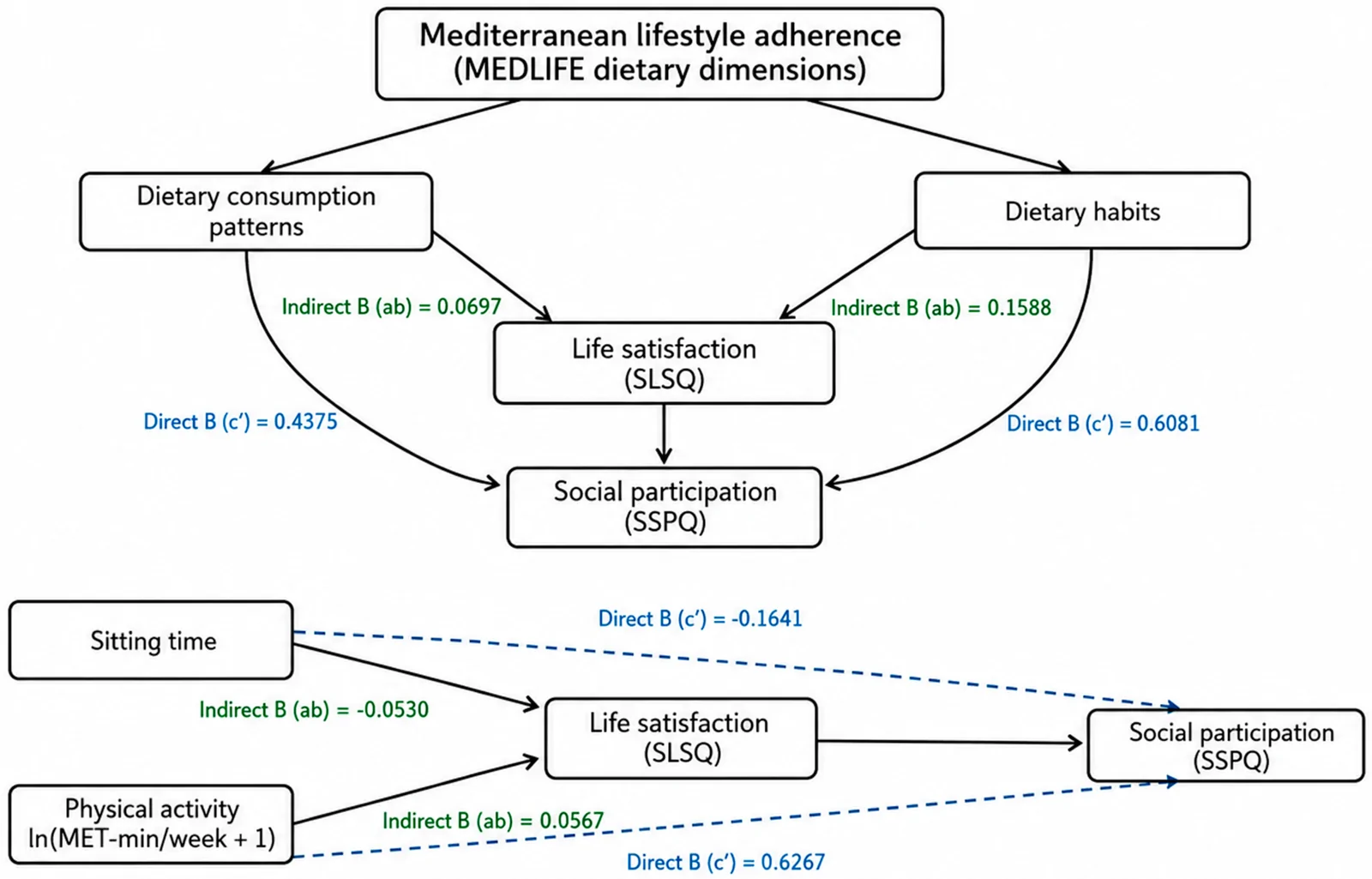
Exploratory statistical indirect association models linking Mediterranean dietary dimensions and movement behaviours with social participation through life satisfaction. Direct B (c′) represents the exposure–social participation association conditional on life satisfaction and covariates, whereas indirect B (ab) represents the estimated statistical indirect association through life satisfaction. Physical activity was entered as ln(MET-min/week + 1). Arrows represent modelled statistical associations and should not be interpreted as temporal or causal pathways because all variables were assessed cross-sectionally.

The four prespecified exploratory models yielded indirect associations through life satisfaction whose bootstrap 95% CIs excluded zero (Table 2 and Figure 1). Positive indirect associations were observed for MEDLIFE dietary consumption patterns (indirect B = 0.0697, 95% CI: 0.0387 to 0.1028), MEDLIFE dietary habits (B = 0.1588, 95% CI: 0.1152 to 0.2085), and physical activity (B = 0.0567, 95% CI: 0.0301 to 0.0843). Sitting time showed a negative indirect association (B = −0.0530, 95% CI: −0.0762 to −0.0313). Because all variables were measured concurrently, these estimates describe exploratory cross-sectional statistical indirect associations and do not establish mediation, temporal ordering, or causal mechanisms.

## 4. Discussion

The present study examined social participation as the primary outcome within a multidomain analysis of the MEDIET4ALL sample. The primary fully adjusted Model 6 explained approximately 23% of the variance in social participation, with the largest standardized associations observed for age, anxiety, life satisfaction, physical activity, and depression. Mediterranean dietary behaviours, sitting time, and selected sleep characteristics also remained associated with social participation. All 16 Model 6 associations with nominal *p* < 0.05 remained statistically supported after FDR correction, and the principal associations were materially consistent in the country fixed-effects and categorical-coding sensitivity analyses. The findings indicate that social participation was associated with several interrelated sociodemographic, lifestyle, sleep-related, and psychological domains, while most of its variability remained unexplained by the measured variables.

Exploratory analyses identified positive indirect associations involving life satisfaction for both Mediterranean dietary dimensions and physical activity and a negative indirect association for sitting time. These findings suggest interrelationships among lifestyle behaviours, positive well-being, and social participation but, given the cross-sectional design, do not establish temporal or causal pathways.

### 4.1. Sociodemographic and Contextual Correlates of Social Participation

Age showed the strongest negative standardized association with social participation in the primary fully adjusted Model 6, indicating that older participants reported lower participation after adjustment for lifestyle, sleep-related, psychological, and background characteristics. This finding is consistent with evidence linking older age with changes in mobility, retirement, bereavement, health limitations, social networks, and environmental barriers that may constrain participation [1,59,60,61]. Nevertheless, MEDIET4ALL included adults across a broad age range and was not designed specifically as an ageing cohort. Age-related differences may also vary according to family structures, community traditions, and cultural context. Previous MEDIET4ALL analyses identified substantial cross-country variation in social participation [43], and the variance remaining unexplained by Model 6 indicates that additional social, cultural, interpersonal, and environmental factors are likely relevant.

Female sex was also negatively associated with social participation after full adjustment. Possible explanations include differences in time availability, caregiving responsibilities, occupational roles, perceived safety, cultural expectations, and access to leisure or public-space activities. These factors were not measured directly and should therefore not be inferred as mechanisms. Previous MEDIET4ALL analyses demonstrated sex-related variation in Mediterranean lifestyle adherence and related behaviours [45], while the broader literature indicates that participation patterns can vary by sex, age, caregiving role, and residential context [1,61]. Because prior findings concerning sex are not uniform across types of social activity, the present association should be regarded as sample- and context-specific.

Higher education was positively associated with social participation. Educational attainment may correspond to greater social resources, health literacy, civic engagement, and access to organized cultural or community activities [62,63]. MEDIET4ALL studies have also identified education as a correlate of lifestyle behaviours, psychosocial well-being, and health status, emphasizing the relevance of socioeconomic and cognitive resources to lifestyle opportunities [19,34,47]. More broadly, education and socioeconomic position may influence access to and engagement with lifestyle and digital-health initiatives, although these factors are often incompletely captured in intervention research [64].

The employment-status coefficient in the primary model should be interpreted cautiously because the survey codes combine heterogeneous categories. In the indicator-coded sensitivity analysis, students reported higher social participation than employed participants, whereas unemployed, retired, and other or uncategorized participants did not differ significantly from the employed reference group. The student estimate may be compatible with the structured interpersonal opportunities available in educational settings. By contrast, employment, retirement, and unemployment may each provide or restrict social opportunities differently according to time, resources, and context. These possible explanations were not assessed directly, and the category-specific findings were secondary.

The indicator-coded analysis also clarified the living-environment association: rural participants reported lower social participation than urban participants, whereas the suburban–urban comparison was not statistically supported. Although rural communities may support strong informal interpersonal ties, participation measured by the SSPQ may also depend on transport, digital infrastructure, educational institutions, leisure facilities, and access to organized activities. Rural settings may therefore support some forms of informal connectedness while limiting structured participation opportunities [20,22]. Recent evidence similarly suggests that urban–rural differences may partly reflect unequal access to community infrastructure and activities [61]. Previous MEDIET4ALL findings also demonstrated variation in social participation across national contexts [43]. The primary purpose of the indicator-coded sensitivity analysis was nevertheless to establish that the principal lifestyle and psychological associations were not materially dependent on the original coding of heterogeneous categorical covariates.

### 4.2. Mediterranean Dietary Behaviours, Movement Patterns, and Social Participation

Both MEDLIFE dietary consumption patterns and Mediterranean dietary habits were positively associated with social participation in Model 6. This finding is compatible with the broader Mediterranean lifestyle framework, in which dietary behaviours occur within social, cultural, and behavioural routines rather than representing nutritional choices alone. Traditional Mediterranean living incorporates shared meals, conviviality, family- and community-based food practices, and everyday social interaction [12,15,16,17]. The observed associations may therefore reflect, in part, the social context of dietary practices, including shared meals, food traditions, and family routines. However, these contexts were not measured directly, and the cross-sectional data cannot determine whether dietary practices relate to subsequent participation, participation facilitates such dietary practices, or both reflect shared determinants. The present findings complement previous MEDIET4ALL analyses demonstrating that Mediterranean lifestyle adherence is related to sociodemographic, behavioural, psychological, and contextual characteristics rather than diet alone [19]. They are also consistent with country-level findings showing that social participation varies across countries and forms part of broader lifestyle profiles [43]. Importantly, the present analysis used the two dietary MEDLIFE domains rather than the total score, thereby excluding the social-interaction and conviviality items contained in the third MEDLIFE domain. This reduces direct construct overlap between the dietary predictors and SSPQ, although it does not eliminate the possibility that dietary and social practices share unmeasured contextual determinants.

The dietary-habits association was slightly stronger than the association involving dietary consumption patterns. Consumption patterns primarily describe food-group intake, whereas the dietary-habits domain captures behavioural features such as moderation, beverage choices, whole-grain preference, and limiting snacks, salt, and sugar-sweetened beverages. Its somewhat stronger association may indicate that the behavioural organization of eating is more closely related to social routines than food intake composition alone. This interpretation is compatible with the Mediterranean lifestyle concept, in which how and in what context people eat may be socially relevant alongside what they consume [12,13,17]. Nevertheless, the difference in coefficient magnitude was not itself subjected to a formal comparative test and should not be overinterpreted.

Sensitivity analyses provided further contextual information. The association involving dietary consumption patterns did not differ by region or sex, and neither dietary dimension showed sex-specific effect modification. In contrast, the positive dietary-habits association was stronger among participants from Mediterranean countries. This secondary interaction may be compatible with greater cultural and social embedding of Mediterranean eating practices in Mediterranean settings, where shared meals, conviviality, family food traditions, and socially situated eating have historically been prominent [12,44,48,65]. However, cultural embedding was not measured directly. The result should therefore be regarded as hypothesis-generating evidence of contextual heterogeneity rather than confirmation that Mediterranean cultural context modifies the association, and it requires independent replication. The country fixed-effects analysis yielded materially consistent estimates for the principal lifestyle and psychological correlates, indicating that these associations were not dependent solely on adjustment for the broad regional classification. This strengthens confidence in the overall pattern of individual-level associations but does not establish that the relationships are identical across countries or identify the country-level factors responsible for contextual differences.

Physical activity showed one of the largest positive standardized associations with social participation, whereas daily sitting time was negatively associated. This pattern is consistent with the socially embedded character of some movement behaviours. Sport, active commuting, walking groups, community recreation, and family-based activities may provide opportunities for interpersonal contact, while social networks may support continued activity through shared routines and encouragement [20,21,23]. Conversely, extended sitting may coincide with occupational demands, screen exposure, limited mobility, or fewer opportunities for participation. The survey did not distinguish socially organized from individual physical activity or characterize the context of sitting, so these explanations remain hypothetical.

These findings complement previous MEDIET4ALL analyses in which social participation was positively associated with physical activity and inversely associated with sitting time when movement behaviours were treated as outcomes [46]. Examining the associations from both analytical perspectives supports the presence of interrelationships among movement and social behaviours but does not establish their temporal direction. Sedentary behaviour should also not be considered simply the absence of physical activity, because it may have distinct occupational, transport-related, technological, and social correlates [24,25,26,27]. Community walking, group activity, active breaks, and other socially situated movement opportunities can therefore be considered candidates for prospective intervention testing, rather than interventions whose effectiveness is demonstrated by the present results [21,66].

### 4.3. Sleep, Psychological Well-Being, and Distress in Relation to Social Participation

Longer sleep duration and better subjective sleep quality were positively associated with social participation. These findings are consistent with literature relating adequate and restorative sleep to daytime functioning, emotional regulation, motivation, and social engagement [28,29,30]. One possible interpretation is that more favourable sleep supports the energy and psychological readiness needed for participation. Conversely, social participation may correspond to more structured daily routines or other aspects of well-being related to sleep. The present design cannot distinguish among these possibilities. The positive association between insomnia severity and social participation is counterintuitive and warrants particular caution, given the established associations of insomnia with fatigue, poorer mental health, and impaired functioning [30,31,67]. Statistical suppression is one possible explanation because Model 6 simultaneously included subjective sleep quality, sleep duration, life satisfaction, depression, anxiety, stress, and other correlated variables. Alternatively, socially active schedules, evening activities, or unmeasured contextual characteristics could coexist with greater insomnia symptoms in some participants. These explanations were not tested and should not be treated as established mechanisms. Sleep latency and sleep efficiency showed substantial multicollinearity, reflecting their conceptual and mathematical relationships with the other sleep indicators. Nevertheless, excluding both variables produced negligible changes in model fit and in the principal regression estimates, indicating that the main association pattern was not materially driven by their inclusion. Previous MEDIET4ALL sleep analyses showed that sleep characteristics are embedded within a broader lifestyle and psychological framework involving life satisfaction, psychological distress, Mediterranean dietary dimensions, and social participation [48]. The present findings complement that work by treating social participation as the outcome, but the reciprocal analytical perspectives remain correlational and hypothesis-generating.

Psychological variables were among the strongest correlates of social participation. Life satisfaction showed a positive association, whereas depressive symptoms showed a negative association. These findings are consistent with evidence linking social connectedness and participation with subjective well-being, belonging, meaningful activity, and lower depressive burden [6,8,35,36,40]. Depressive symptoms may coincide with anhedonia, fatigue, social withdrawal, and reduced motivation, while social engagement may itself relate to well-being and perceived support. Population-based and meta-analytic evidence likewise supports an association between lower social participation and greater depressive burden [3,10,61,68]. These explanations remain potentially reciprocal and cannot be ordered temporally using the present data. The positive anxiety association requires a more nuanced interpretation. Although anxiety is commonly related to avoidance and impaired social Kylie functioning, it may also coexist with greater interpersonal sensitivity, social obligations, or exposure to socially demanding situations. Simultaneous adjustment for depression, anxiety, and stress may also have produced suppression because these constructs overlap conceptually and statistically. After full adjustment, depression was negatively associated with participation, stress was not statistically supported, and anxiety was positively associated. A similar need for caution arose in previous MEDIET4ALL movement-behaviour analyses, in which anxiety and stress showed differing associations [46]. The present finding should not be interpreted as indicating that anxiety is beneficial; it may instead reflect psychological heterogeneity within the multivariable model. Future research should examine whether it differs by symptom profile, age, sex, country, or type of social participation.

Technology use was not independently associated with social participation in Model 6. The three-item STuQL may combine technology used for social communication, Venus lifestyle self-management, and other purposes that have different relationships with participation. A null adjusted association therefore does not demonstrate that digital tools are irrelevant to social engagement. Future studies should distinguish active social communication, passive screen exposure, and technology used to support dietary or physical activity behaviours [64].

### 4.4. Life Satisfaction in the Indirect Associations Between Lifestyle Behaviours and Social Participation

The exploratory analyses identified positive indirect associations involving life satisfaction for both MEDLIFE dietary dimensions and physical activity, and a negative indirect association for sitting time. The dietary findings are compatible with a broader configuration in which food-related practices, evaluative well-being, and social engagement are statistically interrelated. Mediterranean dietary behaviours may be embedded in shared meals, regular eating routines, cultural identity, enjoyment, and social connection, each of which has been related to subjective well-being [12,13,17,69]. The numerically larger indirect estimate for dietary habits than for dietary consumption patterns may be consistent with the behavioural and routine-based character of that domain, although the two indirect estimates were not formally compared.

The movement-related estimates showed complementary directions. Greater physical activity was positively linked to social participation through life satisfaction in the specified models, whereas sitting time showed a negative indirect association. These patterns are consistent with evidence connecting physical activity with positive well-being and social opportunities and connecting sedentary behaviour with less favourable well-being and constrained behavioural engagement [20,21,22,23,24,25,26,27,70]. They also complement previous MEDIET4ALL findings in which social participation was related positively to physical activity and inversely to sitting time [46].

These analyses do not demonstrate that dietary or movement behaviours influence social participation by changing life satisfaction. Alternative sequences are equally plausible: greater social participation may relate to higher life satisfaction and provide opportunities for shared meals or physical activity, while individuals with greater well-being may be more socially and behaviourally engaged. Shared health, socioeconomic, cultural, social, or environmental determinants may also contribute to all variables. Moreover, some physical activities are inherently social, and the observed relationships may partly reflect activity context rather than an indirect psychological process. These alternatives were not directly assessed. Life satisfaction should therefore be regarded as a theoretically informed statistical linking construct, and the indirect estimates as exploratory, hypothesis-generating relationships rather than causal mediation.

### 4.5. Integration Within the MEDIET4ALL Framework and Public-Health Implications

The present study adds to the MEDIET4ALL evidence base by positioning social participation as the primary outcome within an integrated lifestyle analysis. Earlier studies examined Mediterranean lifestyle adherence, movement behaviours, sleep, psychological well-being, or health status as outcomes and demonstrated their relationships with social participation and other contextual factors [19,34,43,44,45,46,47,48]. The current analysis reverses that perspective and shows that dietary behaviours, movement patterns, selected sleep indicators, and psychological functioning are jointly associated with social participation. The country fixed-effects sensitivity analysis further indicated that the principal associations were not attributable solely to differences captured by the broad regional indicator.

From a public-health perspective, the findings identify social participation as a potentially relevant dimension to consider alongside diet, movement behaviours, sleep, and psychological well-being in multidimensional lifestyle-health frameworks. However, no intervention was evaluated, and the data do not demonstrate that modifying any of these domains would increase social participation or improve health outcomes. Shared food-related activities, group physical activity, accessible social opportunities, sleep-health education, and psychological well-being components may be considered candidate elements for prospective development and testing rather than evidence-based prescriptions arising from this study [17,66,71,72].

The regional interaction additionally underscores the potential relevance of sociocultural context. Nevertheless, the Mediterranean versus neighbouring/non-Mediterranean classification is broad, and national culture, family structures, urbanization, transport, socioeconomic resources, and community infrastructure may vary substantially within each regional group. Context-sensitive approaches might therefore require adaptation to local structural and cultural circumstances, but the present study did not test which approaches would be effective. Future intervention research could examine whether accessible community spaces, flexible group activities, or programmes building on existing family and community practices improve social participation in particular settings [4,19,22,34,46,64].

### 4.6. Strengths, Limitations, and Future Directions

This study has several strengths, including its large multinational sample from ten Mediterranean and neighbouring countries, standardized multilingual survey framework, and examination of social participation as the primary outcome within an integrated multidomain model. The hierarchical approach permitted progressive adjustment across sociodemographic, health-related, dietary, movement-related, sleep-related, psychological, and technology-use domains. Complementary analyses assessed collinearity, FDR adjustment, sex- and region-related effect modification, country fixed effects, and alternative indicator coding of categorical covariates. The restricted indirect-association analyses additionally provided a hypothesis-generating examination of life satisfaction as a theoretically specified statistical linking construct.

Several limitations should also be acknowledged. First, the cross-sectional design precludes causal inference and does not establish temporal ordering. This limitation applies to both the primary regression coefficients and the exploratory indirect-association models; reverse or reciprocal relationships and shared determinants remain possible. Second, all variables were self-reported and may be affected by recall bias, social-desirability bias, and measurement error. Although the SSPQ demonstrated acceptable overall internal consistency, reliability varied across the seven language versions, and formal measurement invariance across languages or countries was not assessed. Translation procedures, previous international application, and internal-consistency estimates should therefore not be interpreted as establishing cross-cultural equivalence. Moreover, the SSPQ provides an overall participation score and does not distinguish dimensions such as participation quality, perceived support, loneliness, civic engagement, or informal family interaction. The open, non-probability recruitment strategy also limits the representativeness of the sample within each participating country. Third, multi-category sociodemographic and health-related covariates were represented using their survey codes in the prespecified hierarchical models. Although the indicator-coded sensitivity analysis yielded materially consistent principal associations, the original single-slope coefficients should not be interpreted as category-specific comparisons or as evidence of equal differences between successive categories. Substantial multicollinearity was also observed for sleep latency and sleep efficiency, reflecting overlap among the concurrently modelled sleep dimensions. Neither coefficient was independently supported in Model 6, and excluding both variables produced negligible changes in model fit and in the principal regression estimates. Finally, grouping countries as Mediterranean or neighbouring/non-Mediterranean provided a broad contextual comparison but may obscure considerable cultural, socioeconomic, environmental, and behavioural heterogeneity within and between the two groups. Participants were also nested within ten countries. Although the country fixed-effects sensitivity analysis yielded materially consistent principal associations, the study was not designed to estimate country-level contextual or causal effects, and residual within-country dependence and unmeasured country-specific influences cannot be excluded. Residual confounding by unmeasured individual and contextual factors, including income, occupational demands, family structure, transport access, neighbourhood safety, social support, loneliness, and community infrastructure, also remains possible.

Future longitudinal studies should clarify the temporal relationships among Mediterranean dietary behaviours, movement patterns, sleep, psychological well-being, and social participation. Adequately powered country-specific and intersectional analyses are needed to determine whether the regional variation in the dietary-habits association is reproducible and whether it corresponds to measurable differences in eating practices or social context. Future research should also incorporate more detailed measures of social networks, perceived support, loneliness, community belonging, and participation quality, together with objective movement and sleep indicators and contextual information on neighbourhoods, transport, and community resources. Appropriately designed interventions could subsequently test whether changes in candidate lifestyle or social-engagement components precede changes in social participation and related health outcomes.

## 5. Conclusions

Social participation was associated with multiple lifestyle, psychological, sleep-related, and sociodemographic domains in the multinational MEDIET4ALL sample. Greater social participation was associated with stronger Mediterranean dietary consumption patterns and dietary habits, higher physical activity, lower sitting time, better subjective sleep quality, longer sleep duration, higher life satisfaction, and lower depressive symptoms, while age showed the strongest negative standardized association. The principal associations identified in the fully adjusted model remained statistically supported after FDR correction and materially consistent in complementary robustness analyses, including the country fixed-effects specification. The regional variation in the dietary-habits association and the positive indirect associations involving life satisfaction for both Mediterranean dietary dimensions and physical activity, together with the negative indirect association for sitting time, are exploratory, hypothesis-generating findings that require independent longitudinal confirmation. Because the study was cross-sectional and did not evaluate an intervention, it establishes neither causal direction nor the effectiveness of modifying any identified domain. Nevertheless, the findings support examining social participation as a relevant psychosocial outcome within multidimensional Mediterranean lifestyle research.

## Figures and Tables

**Table 1:** Hierarchical multiple linear regression analyses of social-participation correlates, including the primary fully adjusted Model 6 and exploratory parsimonious Model 7.

Predictor	M1 β	M2 β	M3 β	M4 β	M5 β	M6				M7 β
						B	(95% CI)	β	*p*	
Age	−0.27	−0.23	−0.27	−0.27	−0.25	−0.15	−0.17 to −0.12	−0.22	<0.01	−0.23
Sex	−0.07	−0.05	−0.06	−0.05	−0.05	−1.04	−1.65 to −0.44	−0.05	<0.01	−0.05
Region	−0.02	−0.03	−0.04	−0.04	−0.02	−0.38	−0.98 to 0.23	−0.02	0.22	
Education	0.12	0.12	0.11	0.10	0.10	0.72	0.49 to 0.94	0.09	<0.01	0.10
Employment	0.07	0.07	0.07	0.07	0.06	0.48	0.23 to 0.72	0.06	<0.01	0.06
Marital status	0.01	0.01	0.01	0.02	0.02	0.05	−0.50 to 0.60	0.00	0.86	
Living environment	−0.06	−0.06	−0.06	−0.06	−0.06	−0.75	−1.13 to −0.38	−0.06	<0.01	−0.05
BMI		−0.04	−0.04	−0.02	−0.02	−0.02	−0.09 to 0.04	−0.01	0.45	
Smoking		0.04	0.03	0.03	0.02	0.23	−0.13 to 0.58	0.02	0.21	
Alcohol		0.14	0.14	0.13	0.12	1.77	1.28 to 2.25	0.10	<0.01	0.10
Health status		0.04	0.03	0.03	0.02	0.28	−0.22 to 0.79	0.02	0.27	
MEDLIFE dietary consumption patterns			0.08	0.07	0.06	0.29	0.14 to 0.43	0.06	<0.01	0.06
MEDLIFE dietary habits			0.10	0.08	0.08	0.42	0.23 to 0.60	0.06	<0.01	0.06
Physical activity (log-transformed MET-min/week)				0.15	0.16	0.65	0.53 to 0.77	0.15	<0.01	0.15
Daily sitting time				−0.07	−0.07	−0.17	−0.26 to −0.07	−0.05	<0.01	−0.05
Sleep duration					0.07	0.37	0.11 to 0.62	0.06	<0.00	0.04
Sleep latency					−0.07	−0.02	−0.05 to 0.01	−0.07	0.20	
Sleep efficiency					−0.07	−0.10	−0.26 to 0.06	−0.07	0.22	
Subjective sleep quality					0.13	1.25	0.81 to 1.70	0.10	<0.01	0.10
ISI total score					0.09	0.16	0.09 to 0.22	0.09	<0.01	0.09
SLSQ total score						0.36	0.29 to 0.43	0.16	<0.01	0.16
DASS depression						−0.30	−0.40 to −0.20	−0.15	<0.01	−0.14
DASS anxiety						0.41	0.31 to 0.51	0.19	<0.01	0.20
DASS stress						0.04	−0.06 to 0.15	0.02	0.43	
STuQL total score						−0.08	−0.18 to 0.02	−0.02	0.13	
**No. predictors**	7	11	13	15	20	25				16
**R^2^**	0.105	0.127	0.146	0.171	0.186	0.229				0.227
**Adjusted R^2^**	0.103	0.125	0.143	0.168	0.182	0.224				0.224
**ΔR^2^**	0.105	0.023	0.018	0.025	0.015	0.043				
**F change**	66.917	25.898	43.082	61.290	14.942	44.139	73.422^2^			

B = unstandardized regression coefficient; β = standardized regression coefficient; CI = confidence interval. Model 6 contained the complete prespecified predictor set and was designated as the primary inferential model. Model 7 retained predictors with nominal *p* < 0.05 in Model 6 and is displayed only as an exploratory parsimonious summary; its estimates do not account for model-selection uncertainty. Blank cells indicate that the predictor had not yet been entered into the corresponding hierarchical model or was not retained in Model 7. Multi-category covariates were entered using their original survey codes; indicator-coded results are presented in Supplementary Table S3.

**Table 2:** Bootstrapped exploratory statistical indirect association analyses involving life satisfaction and social participation in the MEDIET4ALL sample.

Statistical Model Specification	Direct Association B (c′)	Indirect Association B (ab)	Bootstrap 95% CI for Indirect Association
A1: MEDLIFE dietary consumption patterns → SLSQ → SSPQ	0.4375	0.0697	[0.0387, 0.1028]
A2: MEDLIFE dietary habits → SLSQ → SSPQ	0.6081	0.1588	[0.1152, 0.2085]
B1: Sitting time → SLSQ → SSPQ	−0.1641	−0.0530	[−0.0762, −0.0313]
B2: Physical activity [ln(MET-min/week +1)] → SLSQ → SSPQ	0.6267	0.0567	[0.0301, 0.0843]

B = unstandardized regression coefficient; CI = confidence interval; SLSQ = Short Life Satisfaction Questionnaire; SSPQ = Short Social Participation Questionnaire. Direct association B (c′) represents the association between each exposure and social participation conditional on life satisfaction and the specified covariates. Indirect association B (ab) represents the estimated cross-sectional statistical indirect association through life satisfaction. Indirect associations were estimated using 5000 bootstrap resamples and were considered statistically supported when the bootstrap 95% CI did not include zero. Physical activity was entered as ln(MET-min/week + 1). All models were adjusted for age, sex, region, education level, employment status, BMI, smoking status, alcohol consumption, and self-reported health status.

## Data Availability

The datasets generated and analyzed during the current study are not publicly available at this time as further analyses are ongoing, and additional publications based on these data are in preparation. Data may be made available upon reasonable request to the corresponding author once all planned analyses and publications are completed.

## Supplementary Materials

The following supporting information can be downloaded at https://www.mdpi.com/article/10.3390/healthcare14183039/s1: Table S1: Collinearity diagnostics for predictors included in the fully adjusted model examining correlates of social participation (SSPQ); Table S2: Region-, sex-, and country-level sensitivity analyses and formal interaction tests for associations with social participation; Table S3: Fully adjusted Model 6 sensitivity analysis using indicator coding for multi-category sociodemographic and health-related covariates. Table S4: Benjamini–Hochberg false-discovery-rate adjustment of predictor associations in the fully adjusted Model 6.

## Abbreviations

The following abbreviations are used in this manuscript:

WHO	World Health Organization
MEDLIFE	Mediterranean Lifestyle Index
GDPR	General Data Protection Regulation
BMI	Body Mass Index
SSPQ	Short Social Participation Questionnaire
IPAQ-SF	International Physical Activity Questionnaire—Short Form
MET-min/week	Metabolic Equivalent Task minutes per week
ISI	Insomnia Severity Index
SLSQ	Short Life Satisfaction Questionnaire
DASS-21	Depression Anxiety Stress Scales—21-item version
DASS	Depression Anxiety Stress Scales
STuQL	Short Technology-Use Questionnaire
CI	Confidence Interval
VIF	Variance Inflation Factor
B	Unstandardized regression coefficient
β	Standardized beta coefficient
*p*	*p*-value
R^2^	Coefficient of determination
Adjusted R^2^	Adjusted coefficient of determination
ΔR^2^	Change in R^2^
F change	Change in F-statistic
