# Supplementary material for: Lifestyle, Sleep, and Psychological Correlates of Social Participation Across Mediterranean and Neighbouring Countries: Findings from the MEDIET4ALL Survey
Source: Healthcare (Basel). 2026 Sep 16;14(18):3039. doi: 10.3390/healthcare14183039 (PMC13606431; doi:10.3390/healthcare14183039)
Supplement: Supplementary file 1 [file healthcare-14-03039-s001.zip › healthcare-4531656-supplementary.pdf]

Supplementary Table S1. Collinearity diagnostics for predictors included in the fully adjusted model examining correlates of social participation (SSPQ)

Predictor	Tolerance	VIF
Age	0.540	1.851
Sex	0.864	1.158
Region	0.902	1.108
Education	0.904	1.106
Employment	0.914	1.094
Marital status	0.643	1.554
Living environment	0.944	1.059
BMI	0.850	1.177
Smoking status	0.922	1.084
Alcohol consumption	0.877	1.140
Self-reported health status	0.825	1.212
MEDLIFE dietary consumption patterns	0.928	1.078
MEDLIFE dietary habits	0.888	1.126
Physical activity [ln(MET-min/week + 1)]	0.922	1.084
Sitting time (hours/day)	0.966	1.036
Sleep duration	0.414	2.418
Sleep latency	0.065	15.441
Sleep efficiency	0.060	16.688
Subjective sleep quality	0.625	1.600
Insomnia Severity Index (ISI)	0.489	2.046
Life satisfaction (SLSQ)	0.765	1.307
Depression (DASS-21)	0.286	3.491
Anxiety (DASS-21)	0.335	2.986
Stress (DASS-21)	0.299	3.348
Technology use (STuQL)	0.989	1.011

VIF = variance inflation factor; tolerance = 1/VIF. Collinearity diagnostics were derived from the fully adjusted regression model (Model 6; n = 4,010). Physical activity was entered as ln(MET-min/week + 1). Most predictors showed acceptable collinearity diagnostics; however, sleep latency and sleep efficiency exhibited elevated VIF values, reflecting substantial overlap among conceptually and mathematically related sleep indicators entered simultaneously.

Note: In a supplementary robustness model excluding sleep latency and sleep efficiency, overall model fit and principal predictor estimates remained materially unchanged ($R^2 = 0.229$; adjusted $R^2 = 0.224$)

Model specification	R^2	Adjusted R^2
Full M6	.22918	.22434
Excluding sleep latency	.22887	.22422
Excluding sleep efficiency	.22888	.22424
Excluding both	.22887	.22442

Table S2: Region-, sex-, and country-level sensitivity analyses and formal interaction tests for associations with social participation

MEDLIFE dietary dimension	Regional group	n	B	β	SE	p	95% CI
**Panel A. Region-specific estimates**							
Dietary consumption patterns	Mediterranean	1,416	0.254	0.046	0.130	.051	−0.001 to 0.509
	Neighbouring/non-Mediterranean	2,594	0.277	0.055	0.092	.003	0.097 to 0.457
Dietary habits	Mediterranean	1,416	0.861	0.130	0.159	<.001	0.549 to 1.172
	Neighbouring/non-Mediterranean	2,594	0.176	0.027	0.120	.142	−0.059 to 0.410
**Panel B. Sex-specific estimates**							
Dietary consumption patterns	Men	1,625	0.240	0.047	0.113	.034	0.019 to 0.461
	Women	2,385	0.307	0.059	0.099	.002	0.112 to 0.502
Dietary habits	Men	1,625	0.513	0.078	0.145	<.001	0.228 to 0.798
	Women	2,385	0.333	0.052	0.127	.009	0.085 to 0.582
**Panel C. Formal interaction term**							
Dietary consumption patterns × Region			−0.108	−0.036	0.153	.478	−0.408 to 0.191
Dietary habits × Region			−0.582	−0.152	0.187	.002	−0.949 to −0.216
Dietary consumption patterns × Sex			0.066	0.021	0.147	.652	−0.221 to 0.353
Dietary habits × Sex			−0.019	−0.005	0.184	.917	−0.380 to 0.342
**Panel D. Country fixed-effects sensitivity analysis**							
MEDLIFE dietary consumption patterns			0.199	0.038	0.075	.008	0.052 to 0.346
MEDLIFE dietary habits			0.435	0.067	0.095	<.001	0.249 to 0.621
Physical activity [ln(MET-min/week + 1)]			0.743	0.174	0.063	<.001	0.621 to 0.866
Sitting time			−0.147	−0.044	0.047	.002	−0.238 to −0.055
Life satisfaction (SLSQ)			0.361	0.162	0.035	<.001	0.292 to 0.430
Depression (DASS-21)			−0.282	−0.141	0.052	<.001	−0.384 to −0.181
Anxiety (DASS-21)			0.402	0.189	0.051	<.001	0.302 to 0.502

B = unstandardized regression coefficient; β = standardized regression coefficient; SE = standard error; CI = confidence interval. Mediterranean countries were coded as 1 and neighbouring/non-Mediterranean countries as 2; men were coded as 1 and women as 2. Subgroup-specific estimates in Panels A and B were obtained by repeating the fully adjusted Model 6 within each stratum, with the corresponding stratification variable omitted. Formal effect modification in Panel C was evaluated in the pooled sample by adding individual interaction terms between the mean-centred MEDLIFE dietary dimension and region or sex while retaining the corresponding main effects and all covariates included in Model 6. Differences in subgroup-specific statistical significance were not interpreted as evidence of effect modification; interpretation was based on the formal interaction terms. In Panel D, the broad regional

indicator was replaced by country-specific indicator variables (one country omitted as the reference) to evaluate robustness of the principal individual-level associations to country-specific contextual heterogeneity. The country fixed-effects analysis was used as a sensitivity analysis and was not intended to estimate between-country effects or variance.

Table S3. Fully adjusted Model 6 sensitivity analysis using indicator coding for multi-category covariates

Predictor	B	β	SE	p	95% CI
Constant	33.287	—	7.430	<.001	18.720 to 47.854
Age (years)	−0.097	−0.149	0.015	<.001	−0.126 to −0.068
Female sex (reference: male)	−0.981	−0.048	0.307	.001	−1.583 to −0.378
Mediterranean region (reference: neighbouring/non-Mediterranean)	−0.362	−0.017	0.312	.246	−0.973 to 0.250
Education: high school/professional degree	−1.872	−0.089	0.670	.005	−3.184 to −0.559
Education: bachelor's degree	0.347	0.017	0.671	.605	−0.968 to 1.663
Education: master's/doctorate degree	0.755	0.031	0.704	.284	−0.626 to 2.136
Employment: unemployed	−0.763	−0.024	0.478	.111	−1.701 to 0.175
Employment: student	2.888	0.125	0.426	<.001	2.053 to 3.724
Employment: retired	−0.674	−0.019	0.622	.278	−1.894 to 0.545
Employment: uncategorized/other	−0.258	−0.005	0.766	.736	−1.761 to 1.244
Marital status: married/living as a couple	−0.156	−0.008	0.372	.676	−0.885 to 0.574
Marital status: widowed/divorced/separated	1.240	0.032	0.622	.046	0.020 to 2.460
Living environment: suburban	−0.458	−0.018	0.372	.217	−1.187 to 0.270
Living environment: rural	−1.492	−0.054	0.401	<.001	−2.279 to −0.705
Body mass index	0.015	0.007	0.032	.638	−0.047 to 0.077
Smoking: cigarette smoker	−0.093	−0.004	0.369	.802	−0.816 to 0.631
Smoking: shisha smoker	−0.023	−0.001	0.645	.972	−1.286 to 1.241
Alcohol consumption: category 2	0.995	0.040	0.359	.006	0.290 to 1.700
Alcohol consumption: category 3	4.469	0.078	0.811	<.001	2.880 to 6.058

Predictor	B	β	SE	p	95% CI
Alcohol consumption: category 4	6.714	0.077	1.242	<.001	4.279 to 9.148
Self-reported health: at risk	−0.483	−0.018	0.404	.231	−1.274 to 0.308
Self-reported health: with disease	−0.249	−0.006	0.570	.662	−1.366 to 0.867
MEDLIFE dietary consumption patterns	0.292	0.056	0.074	<.001	0.147 to 0.438
MEDLIFE dietary habits	0.458	0.071	0.095	<.001	0.271 to 0.644
Physical activity [ln(MET-min/week + 1)]	0.619	0.145	0.061	<.001	0.498 to 0.739
Daily sitting time	−0.184	−0.056	0.047	<.001	−0.275 to −0.093
Sleep duration	0.409	0.069	0.128	.001	0.159 to 0.659
Sleep latency	−0.026	−0.087	0.016	.108	−0.058 to 0.006
Sleep efficiency	−0.121	−0.086	0.079	.126	−0.277 to 0.034
Subjective sleep quality	1.202	0.094	0.224	<.001	0.763 to 1.641
ISI total score	0.147	0.085	0.034	<.001	0.080 to 0.214
Life satisfaction (SLSQ)	0.361	0.162	0.035	<.001	0.292 to 0.431
Depression (DASS-21)	−0.292	−0.146	0.052	<.001	−0.393 to −0.191
Anxiety (DASS-21)	0.395	0.186	0.051	<.001	0.296 to 0.495
Stress (DASS-21)	0.056	0.026	0.053	.296	−0.049 to 0.160
STuQL total score	−0.081	−0.022	0.051	.112	−0.181 to 0.019

Note. B = unstandardized regression coefficient; β = standardized regression coefficient; SE = standard error; CI = confidence interval; ISI = Insomnia Severity Index; SLSQ = Short Life Satisfaction Questionnaire; STuQL = Short Technology-use Questionnaire. SSPQ-L total score was the dependent variable. Binary covariates were entered as indicators, and all multi-category sociodemographic and health-related covariates were represented by $k - 1$ indicator variables. Education reference: no schooling completed; Employment reference: employed; Marital-status reference: single; Living-environment reference: urban; Smoking reference: non-smoker; Alcohol reference: category 1 (no alcohol consumption); Health-status reference: healthy. Physical activity was entered as ln(MET-min/week + 1). N = 4,010; number of fitted predictor coefficients = 36; $R^2 = 0.246$; adjusted $R^2 = 0.239$.

Table S4: Benjamini–Hochberg false-discovery-rate adjustment of predictor associations in the fully adjusted Model 6

Predictor	B	β	Nominal p	FDR-adjusted q	Retained in Model 7
Age	−0.147	−0.224	<.001	<.001	Yes
Sex	−1.044	−0.051	<.001	.001	Yes
Region	−0.377	−0.019	.221	.263	No
Education	0.716	0.093	<.001	<.001	Yes
Employment	0.475	0.056	<.001	<.001	Yes
Marital status	0.047	0.003	.865	.865	No
Living environment	−0.754	−0.056	<.001	<.001	Yes
BMI	−0.024	−0.013	.449	.467	No
Smoking status	0.229	0.019	.208	.263	No
Alcohol consumption	1.767	0.105	<.001	<.001	Yes
Self-reported health status	0.282	0.017	.271	.308	No
MEDLIFE dietary consumption patterns	0.286	0.055	<.001	<.001	Yes
MEDLIFE dietary habits	0.417	0.064	<.001	<.001	Yes
Physical activity [ln(MET-min/week + 1)]	0.646	0.152	<.001	<.001	Yes
Sitting time	−0.166	−0.050	<.001	.001	Yes
Sleep duration	0.365	0.062	.004	.007	Yes
Sleep latency	−0.021	−0.067	.202	.263	No
Sleep efficiency	−0.099	−0.071	.216	.263	No
Subjective sleep quality	1.253	0.098	<.001	<.001	Yes
Insomnia Severity Index	0.155	0.090	<.001	<.001	Yes
Life satisfaction	0.356	0.160	<.001	<.001	Yes
Depression	−0.298	−0.149	<.001	<.001	Yes
Anxiety	0.413	0.194	<.001	<.001	Yes
Stress	0.042	0.021	.432	.467	No
Technology use	−0.078	−0.023	.132	.194	No

B = unstandardized regression coefficient; β = standardized regression coefficient; FDR = false discovery rate. Benjamini–Hochberg FDR correction was applied across all 25 predictor tests included in the fully adjusted Model 6. Model 7 was derived by retaining predictors that were nominally significant in Model 6; all 16 predictors retained in Model 7 also remained statistically supported after FDR correction ($q < .05$). FDR adjustment was treated as a multiplicity/stability analysis and was not used to redefine the prespecified hierarchical modelling sequence.
